# Kaiso phosphorylation at threonine 606 leads to its accumulation in the cytoplasm, reducing its transcriptional repression of the tumour suppressor *CDH1*

**DOI:** 10.1002/1878-0261.13292

**Published:** 2022-07-28

**Authors:** Wei Tian, Hongfan Yuan, Sisi Qin, Wensu Liu, Baozhen Zhang, Liankun Gu, Jing Zhou, Dajun Deng

**Affiliations:** ^1^ Key Laboratory of Carcinogenesis and Translational Research (Ministry of Education/Beijing), Division of Cancer Etiology Peking University Cancer Hospital and Institute Beijing China

**Keywords:** 14‐3‐3 proteins, AKT1, cancer, Kaiso, P120ctn, phosphorylation, transcription factor

## Abstract

It is well known that the Kaiso protein (encoded by the *ZBTB33* gene) is a transcription factor, and Kaiso–P120ctn [P120 catenin (CTNND1)] interaction increases the translocation of Kaiso from the nucleus into the cytoplasm. However, the regulatory mechanisms of Kaiso compartmentalisation are far from clear. Here, we reported that RAC‐alpha serine/threonine‐protein kinase (AKT1) could phosphorylate threonine residue 606 (T606) within the RSSTIP motif of Kaiso in the cytoplasm. The T606‐phosphorylated Kaiso (pT606‐Kaiso) could directly bind to 14‐3‐3 family proteins, and depletion of T606 phosphorylation by T606A mutation abolished most of the Kaiso–14‐3‐3 binding. In addition, the Kaiso–P120ctn interaction was essential for pT606‐Kaiso accumulation in the cytoplasm. Notably, enforced stratifin (*14‐3‐3σ*; *SFN*) overexpression could increase pT606‐Kaiso accumulation in the cytoplasm and de‐repress the transcription of Kaiso target gene cadherin 1 (*CDH1*), which is a tumour suppressor. Decreased amounts of both pT606‐Kaiso and CDH1 proteins were frequently observed in human gastric cancer tissues compared to paired normal controls. The mRNA levels of *14‐3‐3σ* and Kaiso target gene *CDH1* showed highly significant positive correlations in both human normal tissues and cancer cell lines by bioinformatics analyses. Furthermore, Kaiso T606A mutant (unable to be phosphorylated) significantly increased the migration and invasion of cancer cells *in vitro* and promoted the growth of these cells *in vivo*. In conclusion, Kaiso could be phosphorylated at T606 by AKT1 and pT606‐Kaiso accumulates in the cytoplasm through binding to 14‐3‐3/P120ctn, which de‐represses the Kaiso target gene *CDH1* in normal tissues. Decreased Kaiso phosphorylation might contribute to the development of gastrointestinal cancer. The status of Kaiso phosphorylation is a determinant factor for the role of Kaiso in the development of cancer.

## Graphical abstract

Deregulated cellular localisation of the transcriptional factor Kaiso was described to play a role in gastrointestinal cancer. Here we report that phosphorylation of Kaiso at T606 by AKT1‐mediated binding to Stratifin/P120ctn and accumulation of Kaiso in the cytoplasm. Translocation of Kaiso from the nucleus resulted in transcriptional de‐repression of the *CDH1* gene and inhibited oncogenesis. Our data indicate that decreased Kaiso phosphorylation may contribute to the development of gastrointestinal cancer.

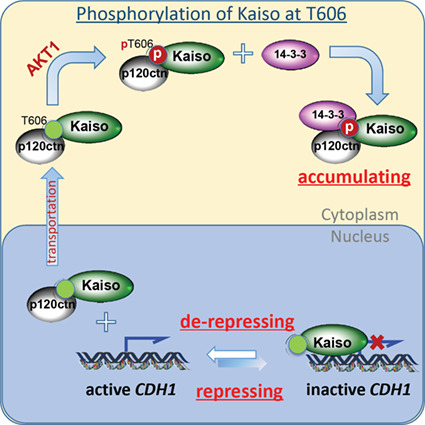

**Abbreviations**
BSA: bovine serum albumin
CCLE: Cancer Cell Line Encyclopaedia
CIAP: calf intestinal alkaline phosphatase
Co‐IP: co‐immunoprecipitation
CY5: Cyanine5
DAPI: 4′; 6‐diamidino‐2‐phenylindole
FITC: fluorescein isothiocyanate
GTEx: Genotype‐Tissue Expression
IP: immunoprecipitation
IHC: immunohistochemical
KBS: Kaiso binding sequence
NOD‐SCID mice: Non‐obese diabetic/severe combined immunodeficient mice
P120ctn: P120 catenin (CTNND1)
pAKT‐Sub: phosphorylated AKT substrate
PBS: Phosphate‐buffered saline
Phos‐tag: Phos‐tag molecular binds specifically to phosphate group in proteins via metal ions
pT606: T606‐phosphorylated
T606A: replacement of the 606th amino acid residue threonine with alanine
WT: wild‐type

## 1 Introduction

Kaiso protein encoded by the *ZBTB33* gene is a classic transcription repressor containing a zinc‐finger domain and a BTB/POZ domain [1]. The zinc‐finger domain of Kaiso can bind to both methylated CGCG‐containing sequences and TpG‐containing specific binding sequences within Kaiso target genes, and the BTB/POZ domain can further recruit the complex of NCoR1 corepressor and histone deacetylases to target genes and repress their transcription in the nucleus [2, 3, 4, 5, 6]. Recent studies show that Kaiso may also act as a transcription activator in the promoter context‐dependent manner [7, 8].

As a transcription repressor, Kaiso controls the cell cycle through repressing *CCND1* and *CCNE1* expression, affects Notch signalling pathway in intestinal cells through targeting *DLL1* and *JAG1* promoter, and inhibits the proliferation and invasion of tumour cells through downregulating *MMP7*, *MTA2* and other genes [9, 10, 11, 12]. Kaiso also represses *CDH1* and *CDKN2A* expression [5, 13]. Interestingly, it has been reported that the amount of nuclear Kaiso, but not total Kaiso, is correlated with the invasion or prognosis of cancers [14, 15]. *Kaiso*‐deficient mice show resistance to intestinal cancer [16]. Apparently, the expression and subcellular location states of Kaiso determine its normal functions and roles in cancer development.

Kaiso is also present in the cytoplasm, which regulates WNT‐related pathway through interacting with P120ctn (CTNND1) protein (Fig. S1) [1, 17]. Subcellular locations of Kaiso are different between cultured cells and tissues [13, 18]. The P120ctn–Kaiso complexes could shift from the nucleus to the cytoplasm [19]. Kaiso nuclear‐cytoplasmic trafficking could be affected by environmental factors, such as cigarette smoke, through MUC1 and P120ctn binding [20]. However, detailed regulation machinery for the compartmentalisation of Kaiso remains far from clear. Phosphorylation signal of Kaiso has been detected in proteomic mass spectrometry analysis [21, 22]. It is unknown whether Kaiso is a true phosphorylation target protein and the phosphorylation affects its compartmentalisation.

The 14‐3‐3 proteins are originally identified in the brain [23]. There are seven human 14‐3‐3 isoforms (α/β, ε, η, δ/γ, τ, ζ and σ). These 14‐3‐3 isoforms are homologous with approximately 50% similarity and capable of forming either homo‐ or hetero‐dimers [24, 25, 26, 27]. The 14‐3‐3 proteins family has been implicated as a key regulator in signal transduction events [28]. Among the family members, 14‐3‐3γ and 14‐3‐3σ (SFN) have been confirmed to play important roles in cancer development [29, 30, 31, 32].

In the present study, we characterised the status of phosphorylation at Thr‐606 (T606) within the RSSTIP motif of Kaiso (pT606‐Kaiso) and the corresponding kinase. Biological and pathological functions of phosphorylated Kaiso were studied in detail and validated in human cancer tissues and animal models. We found, for the first time, that the T606 phosphorylation is a turn‐off switch for Kaiso to function as a transcription factor and the decreased level of phosphorylation of Kaiso could enhance its oncogenic role in cancer development.

## 2 MATERIALS and METHODS

### 2.1 Cell lines and culture

The gastric cancer cell lines MGC803, BGC823, and SGC7901 were kindly provided by Dr. Yang Ke at Peking University Cancer Hospital; MKN45 cell line was purchased from the National Infrastructure of Cell Line Resource (Beijing, China). Breast cancer cell line MCF7 was provided by Dr. Yang Ke at Peking University Cancer Hospital; prostate cancer cell line PC3, purchased from Cell Line Bank, Chinese Acad Med Sci; lung cancer cell line H1299, provided by Dr. Chenchao Shou at Peking University Cancer Hospital; colon cancer cell line RKO, provided by Dr. Guoren Deng at University of California, and pancreas cancer cell line PANC1, provided by Dr. Yuanjia Chen at Peking Union Medical College Hospital. MGC803, BGC823, SGC7901, MKN45, H1299, RKO and PANC1 cells were cultured in RPMI 1640 medium containing 10% FBS. MCF7 cells were cultured in DMEM with 10% FBS and PC3 cells were cultured in F12 medium with 10% FBS. All these cells were cultured in the above medium containing 100 U·mL^−1^ penicillin/streptomycin (Life Technologies, Carlsbad, CA, USA) at 37 °C in a humidified incubator with 5% CO_2_. These cell lines were tested and authenticated by Beijing JianLian Gene Technology Co. before use. Short tandem repeat (STR) patterns were analysed using GoldeneyeTM20A STR Identifier PCR Amplification Kit.

### 2.2 Gastric carcinoma tissues and ethical issue

Gastric cancer tissues and the paired normal surgical margin tissues were collected from 12 patients at Peking University Cancer Hospital from 2000 to 2001 and stored at −80 °C. The Institutional Review Board of the Peking University Cancer Hospital approved the study, which was carried out in accordance with the principles outlined in the Declaration of Helsinki. All patients provided written informed consents.

### 2.3 Plasmids and reagents

The full‐length Kaiso‐coding sequence of the *ZBTB33* gene (chrX:119384607‐119392251, hg19) was amplified from human cDNA of MGC803 cells with a primer set (forward 5′‐attaaactcgaggcatggagagtagaaaactga‐3′ and reverse 5′‐cgcttcgaattcgtttagtaagactctggtattat‐3′), then inserted into *Xho*I and *Eco*RI sites of pEGFP‐C1 vector to generate pEGFP‐C1‐Kaiso expression vector. pEGFP‐C1‐Kaiso‐T606 mutants were obtained by mutation PCRs using a primer set (forward 5′‐gatagatcaagcgctattcctgcaatg‐3′ and reverse 5′‐cattgcaggaatagcgcttgatctatc‐3′) for 606Thr ➔ Ala (T606A) mutation. Plasmid pEBG‐GST‐Kaiso was generated by inserting the full‐length Kaiso coding sequence into *BamH*I and *Not*I sites of pEBG vector.

pcDNA3.1‐HA‐AKT1 vector was purchased from Addgene Co. (#9008, Watertown, MA, USA); pEZ‐M56‐14‐3‐3γ‐mCherry and pEZ‐M56‐14‐3‐3σ‐mCherry vectors were purchased from FulenGen Co. (EX‐T4084‐M56, EX‐T4084‐M98‐5, EX‐C0507‐M98, Guangzhou, China); pENTER‐Flag‐14‐3‐3 isoforms (α/β, ε, η, δ/γ, τ, ζ and σ) were purchased from Vigene Bioscience Co. (CH867785, CH897212, CH845486, CH898602, CH878525, CH824520 and CH890307, Shandong, China); Insulin (P3376, Beyotime, Shanghai, China), IL‐6 (Cat. 200‐06, Proteintech, NJ, USA), EGF (PHG6045, Thermo Fisher Scientific, MA, USA) and MK2206 (HY‐10358, MedChemExpress, Monmouth Junction, NJ, USA) were also used in the study. *P120ctn*‐specific siRNAs (#1: sense 5′‐gaaugugaugguuuaguuuu‐3′ and antisense 5′‐aacuaaaccaucacauucuu‐3′; #2: sense 5′‐uagcugaccuccugacuaauu‐3′ and antisense 5′‐uuagucaggaggucagcuauu‐3′; #3: sense 5′‐ggaccuuacugaaguuauuuu‐3′ and antisense 5′‐aauaacuucaguaagguccuu‐3′) were synthesised by Genepharma Co. (Shanghai, China). The quantitative RT‐PCR primer sequences for detection of the level of *CDH1* mRNA were: forward 5′‐gaacgcattgccacatacac‐3′ and reverse 5′‐gaattcgggcttgttgtcat‐3′ (*T*_m_ = 58 °C). The *Alu* RNA was used as the reference (forward 5′‐gaggctgaggcaggagaatcg‐3′ and reverse 5′‐gtcgcccaggctggagtg‐3′, *T*_m_ = 60 °C), as previously described [33].

### 2.4 Cell transfection

X‐tremeGENE siRNA Transfection Reagent or X‐tremeGENE HP DNA Transfection Reagent (Cat. 04476093001, Cat. 06366236001, Roche, Mannheim, Germany) was used in cell transfection with siRNAs against *P120ctn* (final concentration, 100 nm) or Kaiso or its mutant expression vectors (2 μg/well in 6‐well plates) following manufacturer's instructions. The efficiency of gene overexpression or knockdown was determined 48 or 72 h post‐transfection by western blotting. For stable transfection, G418 was added into the medium to select consistent GFP‐Kaiso expressing MGC803 cells (final concentration, 750 μg·mL^−1^). Flow sorting assay was performed with a FACS Calibur flow cytometer (BD Biosciences, Franklin Lakes, NJ, US) after 48 h cell transfection, then sorted transfected‐SGC7901 cells and MKN45 cells were grown in 6‐well plates under the G418 selection.

### 2.5 Wound healing assay

All cells were seeding in 6‐well plates (five wells per treatment). After reaching 95–100% confluence, the wound healing assays were performed [34]. A pipette tip was used to gently scratch the cell monolayer. After two washes with PBS, the cells were cultured with serum‐free RPMI‐1640 medium. Images of wound healing were captured at different times.

### 2.6 Transwell assays

Transwell assays (three wells per treatment) were performed to determine the migration and invasion of cancer cells [34]. For the migration assay, all cells (2 × 10^4^ cells per chamber) were separately resuspended in 180 μL of serum‐free RPMI 1640 medium and seeded in the upper chambers (8 μm pores; Corning Inc., Corning, NY, USA). For the invasion assay, the upper chamber pre‐coated with Matrigel (BD Biosciences, Franklin Lakes, NJ, USA) was used. Then, cells (4 × 10^4^ cells per chamber) were separately seeding in the upper chambers. After 24–48 h' incubation, these chambers were fixed with 4% paraformaldehyde for 30 min and stained with 0.1% crystal violet. Images of migrating and invading cells were captured using a microscope (Leica DMI4000B, Milton Keynes, Bucks, UK).

### 2.7 Cell proliferation assay

Cell counting kit‐8 (CCK‐8 Kit; C0037, Beyotime, Shanghai, China) was used to detect cell proliferation [35]. Briefly, all cells at the density of 2 × 10^3^ cells per well were seeding in 96‐well plate. Cell proliferation was assessed at 0, 24, 48 and 72 h by adding 10 μL of CCK‐8 solution to each well. After 2 h' incubation with CCK‐8, the absorbance at 450 nm was quantified by a microplate reader (Tecan Infinite M200 PRO, Mannedorf, Switzerland). The average value for these wells was calculated for each treatment and statistically compared with Student *t*‐test.

### 2.8 Xenografts in NOD‐SCID mice

Cells resuspended in 0.1 mL PBS (1 × 10^7^ cells·mL^−1^) were inoculated subcutaneously into the bilateral inguinal of 6 week‐old female NOD‐SCID mice (9 mice per group, 1 × 10^6^ cells per injection; purchased from Beijing Huafukang Biotech, Beijing, China) [36]. Mice were sacrificed on the 28th inoculation day, and xenografts were separated, weighted and photographed. Housing conditions were 25 °C, humidity 45–55%, 1 atm pressure, 12‐h light/12‐h dark cycle, free choice feeding and water *ad libitum*. All research involving animals complied with protocols approved by the Beijing Medical Experimental Animal Care Commission. License Number for Animal Breeding and using Facilities: SYXK (Jing) 2016‐0015.

### 2.9 Subcellular fractionation and de‐phosphorylation treatment

To prepare cytoplasmic and nuclear extracts, small pieces of tissue or cultured cells at 80% confluence were homogenised in ice‐cold buffer CERI of Nuclear and Cytoplasmic Extraction Reagent (7883, Thermo Fisher, MA, USA) with CERII and 1 × EDTA‐free Protease Inhibitor Cocktail (REF04693159001, Roche, Mannheim, Germany) according to the Instruction. Samples were then vortexed, incubated on ice for 20 min and centrifuged at 14 000 ***g*** for 15 min at 4 °C. The supernatants were recovered to obtain the cytosolic extracts. The pellets were washed sequentially with CERI and then incubated in buffer NER on ice and vortexed 15 s every 10 min. After four times of vortex, centrifuging at 14 000 ***g*** for 15 min, the extract was collected as nuclear proteins. The purities of cytoplasmic and nuclear extracts were respectively verified by probing with anti‐β‐Tubulin and anti‐Lamin B antibodies.

For the calf intestinal alkaline phosphatase (CIAP)‐catalysed de‐phosphorylation, the cytoplasmic and nuclear extracts were aliquoted into two centrifuge microtubes. 1 μL of CIAP (p4978, Merck, Darmstadt, Germany) (> 10 U·μL^−1^) of protein was added into one aliquot (30 μL; ratio of protein extracts to 10 × CIAP buffer, 27 : 3) and incubated for 30 min at 37 °C [37, 38]. Bovine serum albumin (BSA) was used as negative control in equal amounts of protein extracts in 1 × CIAP buffer.

### 2.10 Immunoprecipitation and western blotting

Antibodies for Kaiso (sc‐23871, Santa Cruz, Dallas, TX, USA), P120ctn (66208‐1, Proteintech, Chicago, IL, USA), pan‐14‐3‐3 (sc‐629, Santa Cruz), 14‐3‐3 family (α/β, ε, η, δ/γ, τ, ζ and σ) kits (#9769, CST, USA), 14‐3‐3σ (sc‐100638, Santa Cruz), Ser/Thr/Tyr phosphor‐protein (ab15556, Abcam, UK), phosphorylated AKT substrate [(R/K)X(R/K)XX(pT/pS)] (pAKT‐sub; #9611, CST), AKT(#4691, CST), AKT1(AF0045, Beyotime, Shanghai, China), CDH1 (#3195, CST), Lamin B1 (66095‐1, Proteintech), β‐Tubulin (66240‐1, Proteintech), HA (M20003, Abmart, Shanghai, China), Flag (66008‐2, Proteintech), GFP (NB100‐1614, Novus, CO, USA), GST (66001‐1, Proteintech) and GAPDH (660004‐1, Proteintech) were used in the immunoprecipitation (IP) and western blotting analyses.

After being pre‐cleared with protein A/G‐coupled Sepharose beads (Cat. 11134515001 and 11243233001, Roche, Mannheim, Germany) for 2 h, the nuclear or cytoplasmic lysate was immunoprecipitated with mouse anti‐Kaiso antibody or anti‐Phosphoserine/threonine/tyrosine antibody plus protein A/G Sepharose for 8 h at 4 °C. Mouse IgG was used as a negative control. The precipitates were washed six times with lysis buffer, and boiled in 1 × loading buffer. Protein samples were resolved by SDS/PAGE, and electroblotted onto nitrocellulose membranes, which were blocked in 5% skim milk in PBST and probed with antibodies according to the Instruction Manual.

For the western blotting detection of Kaiso and other proteins in matched tumour and normal gastric tissues, about 50 mm^3^ fresh tissues were cut up on ice and dissociated in 500 μL 1 × SDS loading buffer. Protein samples were resolved by SDS/PAGE and detected as described above.

### 2.11 Phos‐tag SDS/PAGE assay

This is a modified SDS/PAGE method based on the novel Phos‐tag [39], which can bind to phosphorylated proteins and decrease their migration speed. Thus, this assay is often used to distinguish dephosphorylated proteins from phosphorylated proteins on the case of that a phosphorylation‐specific antibody is not available. 50 μm Phos‐tag (final concentration, Phos‐tag Acrylamide AAL‐107, WAKO, Japan) and 100 μm MnCl_2_ were mixed to prepare SDS/PAGE gel.

### 2.12 GST pull‐down

The day after pEBG‐GST‐Kaiso transfection, MGC803 cells (in 10 cm dishes, eight dishes per group) were further transfected with pENTER‐Flag‐14‐3‐3 members (α/β, ε, η, δ/γ, τ, ζ and σ) or negative control vector respectively [40]. 48 h post‐transfection, MGC803 cells were harvested and used to prepare lysate with cell lysis buffer with 1 × Protease Inhibitor Cocktail (REF04693159001, Roche, Mannheim, Germany). The lysate was incubated with Glutathione Sepharose beads (20 μL for one group, 17‐0756‐01, GE healthcare, Sweden) at 4 °C overnight. Beads were washed six times with 500 μL cell lysis buffer. After the last centrifuging, the supernatant was removed as clean as possible, and the pellet was suspended in 20 μL 1 × SDS sample buffer. Antibodies for GST‐Kaiso and Flag‐14‐3‐3 family proteins were used to detect the pulled down precipitant.

### 2.13 Co‐immunoprecipitation

After pre‐cleared with protein A/G‐coupled Sepharose beads for 2 h, the soluble proteins from whole cell lysate were immunoprecipitated with anti‐Kaiso (sc‐365428, Santa Cruz) or anti‐GFP (ab290, Abcam) or other antibodies plus protein A/G Sepharose overnight at 4 °C. Mouse IgG or rabbit IgG was used as a negative control. The precipitates were washed six times with lysis buffer, and boiled after 1 × SDS loading buffer was added. Protein samples were resolved by SDS/PAGE, and electroblotted onto nitrocellulose membranes, which were blocked with 5% skim milk in PBST and probed with the interacted protein antibodies. Loading cell number ratio for Input lane to IP lanes was 1 : 100.

### 2.14 Preparation of phosphorylated Kaiso‐specific antibody

To characterise the phosphorylation status of endogenous Kaiso protein, anti‐pT606‐Kaiso polyclonal antibodies were raised in rabbits challenged with the synthesised phosphorylated peptide LSD**RSSpTIP**AM, a sequence corresponding to amino acids 600‐610 of wild‐type Kaiso (Kaiso‐WT), absorbed with non‐phosphorylated peptide LSD**RSSTIP**AM, and enriched by the phosphorylated peptide LSD**RSSpTIP**AM. Peptide synthesis and immunisation of the animals were done by YouKe Co. (Shanghai, China). The non‐phosphorylated peptide was also used to prepare the control polyclonal antibodies against total Kaiso at the same time. The specificity of pT606‐Kaiso and control antibodies against the corresponding peptide (LSDRSSpTIPAM or LSDRSSTIPAM) was detected with ELISA assay.

### 2.15 ELISA analyses for polyclonal antibodies against phosphorylated Kaiso and total Kaiso

Polystyrene plates were coated with 1 μg·mL^−1^ synthetic phosphorylated peptide LSDRSSpTIPAM or non‐phosphorylated peptide LSDRSSTIPAM link‐coupled by BSA in 1 × CBS buffer overnight at 4 °C, respectively, and were washed three times with PBS containing 0.05% Tween‐20. Unbinding sites were blocked with 5% skim milk at room temperature for 2 h. The purified antibodies were added (100 μL/well) and incubated at 37 °C for 1 h. After being washed with 0.05% Tween‐20/PBS, plates were added HRP‐labelled goat anti‐Rabbit IgG (100 μL/well) and incubated at room temperature for 30 min. Peroxidase activity was measured with 0.15 mg·mL^−1^ TMB substrate solution (100 μL/well). After 15 min at room temperature, the reaction was stopped by 2 m H_2_SO_4_ (50 μL/well). Optical density absorbance (OD) at 450 nm was determined using a model 550 microplate reader.

### 2.16 Immunohistochemical staining

Rabbit pT606‐Kaiso polyclonal antibody (1 : 100) and mouse monoclonal antibody for total Kaiso (1 : 50, sc‐23871, Santa Cruz) were used in the immunohistochemical (IHC) analysis. The REAL™ EnVision™ Detection system including anti‐rabbit/mouse IgG secondary antibodies and peroxidase/diaminobenzidine (Dako, Agilent Technologies, Santa clara, CA, USA) was used to visualise the primary antibody‐binding cells according to the manufacturer's protocol. Briefly, paraffin sections (4 μm) were dewaxed and rehydrated in xylene and ethanol. For antigen retrieval these sections were autoclaved for 3 min in 10 mm sodium citrate buffer containing 0.05% Tween‐20 (pH 6.0) for pT606‐Kaiso and in 1 mm EDTA buffer (pH 8.0) for total Kaiso. Then, these sections were immersed in 3% H_2_O_2_ for 10 min to block endogenous peroxidase. Following submerging in 5% BSA (A1933; Sigma Life Science; Merck KGaA) for 60 min, the sections were incubated with the primary antibody overnight at 4 °C. The PBS‐washed sections were then treated with the REAL™ EnVision™ Detection system and counterstained with haematoxylin (0.125%; Zhongshan Jinqiao Biotechnology, Beijing, China) at room temperature for 1 min. Normal rabbit IgG and normal mouse IgG (cat. no. ZDR 5003 and cat. no. ZDR 5006; Zhongshan Jinqiao Biotechnology) were used as negative controls. The former was diluted and incubated as for the pT606‐Kaiso antibody, and the latter was diluted and incubated as for total Kaiso antibody.

### 2.17 Confocal analysis

For mCherry‐14‐3‐3, direct fluorescence was detected with laser confocal microscope assay. MGC803 cells with mCherry‐14‐3‐3 overexpression were rinsed for three times with PBS, fixed with 1% paraformaldehyde in PBS 30 min at 37 °C, punched with 0.5% triton X‐100 for 10 min at 37 °C, incubated with respective primary and secondary antibodies, washed for three times in PBS, counterstained with DAPI (1 μg·mL^−1^) for 5 min, and then examined with Leica SP5 Laser Scanning Confocal Microscopy. The antibodies for pT606‐Kaiso (1 μg·μL^−1^, 1 : 100), for total Kaiso (commercial antibody, 0.2 μg·μL^−1^, 1 : 20, sc‐365428, Santa Cruz, USA) and for P120ctn (1 : 100, 66208‐1, Proteintech) were used as the primary antibodies; the fluorescein isothiocyanate (FITC)‐labelled antibody against rabbit IgG (1 : 100, ab6717, Abcam, UK) and CY5‐labelled antibody against mouse IgG (1 : 100, Cat. No. 072‐02‐18‐06, KPL Gaithersburg, MD, USA) were used as secondary antibodies for observation under Leica SP5 Laser Scanning Confocal Microscope and were analysed with ImageXpress Micro High Content Screening System.

For the detection of Kaiso in tissues, fresh cryostat sections (4 μm) from gastric carcinoma and the paired normal tissues were fixed with 4% paraformaldehyde for 10 min at 37 °C and treated with 0.5% triton X‐100 for 10 min at 37 °C, and then incubated with respective primary, secondary antibodies and DAPI, and then examined as described above.

### 2.18 Publicly available RNA‐Seq, cDNA array and other datasets

The RNA sequencing datasets in Cancer Cell Line Encyclopaedia (CCLE) and Genotype‐Tissue Expression (GTEx) projects were downloaded from official websites (https://sites.broadinstitute.org/ccle and www.gtexportal.org) [41, 42, 43]. All raw data were transferred to Transcripts per kilobase of exon model per Million mapped reads (TPM) using uniform names for each protein‐coding gene in the human genome. The Pearson correlation coefficient (*r*) of genes expression was calculated by r statistical software (version 3.6.1) with a set of procedures [44]. Briefly, raw data were read through ‘read.table’ function, and an array was established to store data by ‘array’ function. Then the Pearson correlation coefficient was calculated by ‘cor’ function, and finally saving the data to files in csv format by the function of ‘write.table’.

## 3 Results

### 3.1 Discovery of phosphorylation of Kaiso in the cytoplasm

**Fig. 1:**
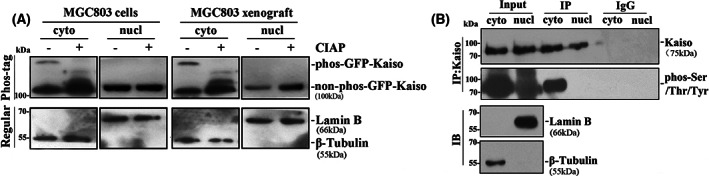
Different phosphorylation states of Kaiso protein in the cytoplasm and nucleus of cells *in vitro* and *in vivo*. (A) The phosphorylation statuses of GFP‐Kaiso stably transfected MGC803 cells and the corresponding xenograft tissues in the Phos‐tag SDS/PAGE analysis. Data are representative of at least two independent experiments. (B) The phosphorylation statuses of endogenous cytoplasmic and nuclear Kaiso in MGC803 cells were validated using anti‐phosphoSer/Thr/Tyr universal antibody. Data are representative of at least two independent experiments.

Transcription factor Kaiso locates not only in the nucleus, but also in the cytoplasm. The regulatory mechanism of Kaiso compartmentalisation is still unknown. Previous study has showed that there might be putative phosphorylation sites within Kaiso by proteomic mass spectrometry analysis (Fig. S1) [21, 22, 45]. Therefore, we wondered if these putative phosphorylation sites can affect Kaiso compartmentalisation. In Phos‐tag SDS/PAGE assay, we found that the cytoplasmic and nuclear Kaiso from GFP‐Kaiso stably transfected MGC803 cells migrated at the same speed in regular SDS/PAGE gel. However, the cytoplasmic Kaiso migrated much slower than the nuclear Kaiso in the Phos‐tag gel, whether these cells were cultured *in vitro* or transplanted into nude mice as a xenograft. No phosphorylated Kaiso was detected with the CIAP de‐phosphorylation (Fig. 1A).

To validate the different phosphorylation states of Kaiso between the cytoplasm and nucleus, an anti‐phosphoserine/threonine/tyrosine universal antibody was then used to precipitate all the phosphorylated proteins and then a Kaiso‐specific antibody was used to visualise the possible phosphorylated Kaiso (Fig. 1B). Again, phosphorylated Kaiso was observed only in the cytoplasmic precipitates, but not in the nuclear counterpart, suggesting that there may be phosphorylation of Kaiso in the cytoplasm. Thus, Kaiso phosphorylation kinase and target site were further characterised in details as described below.

### 3.2 Kaiso is phosphorylated at T606 by AKT1 kinase

**Fig. 2:**
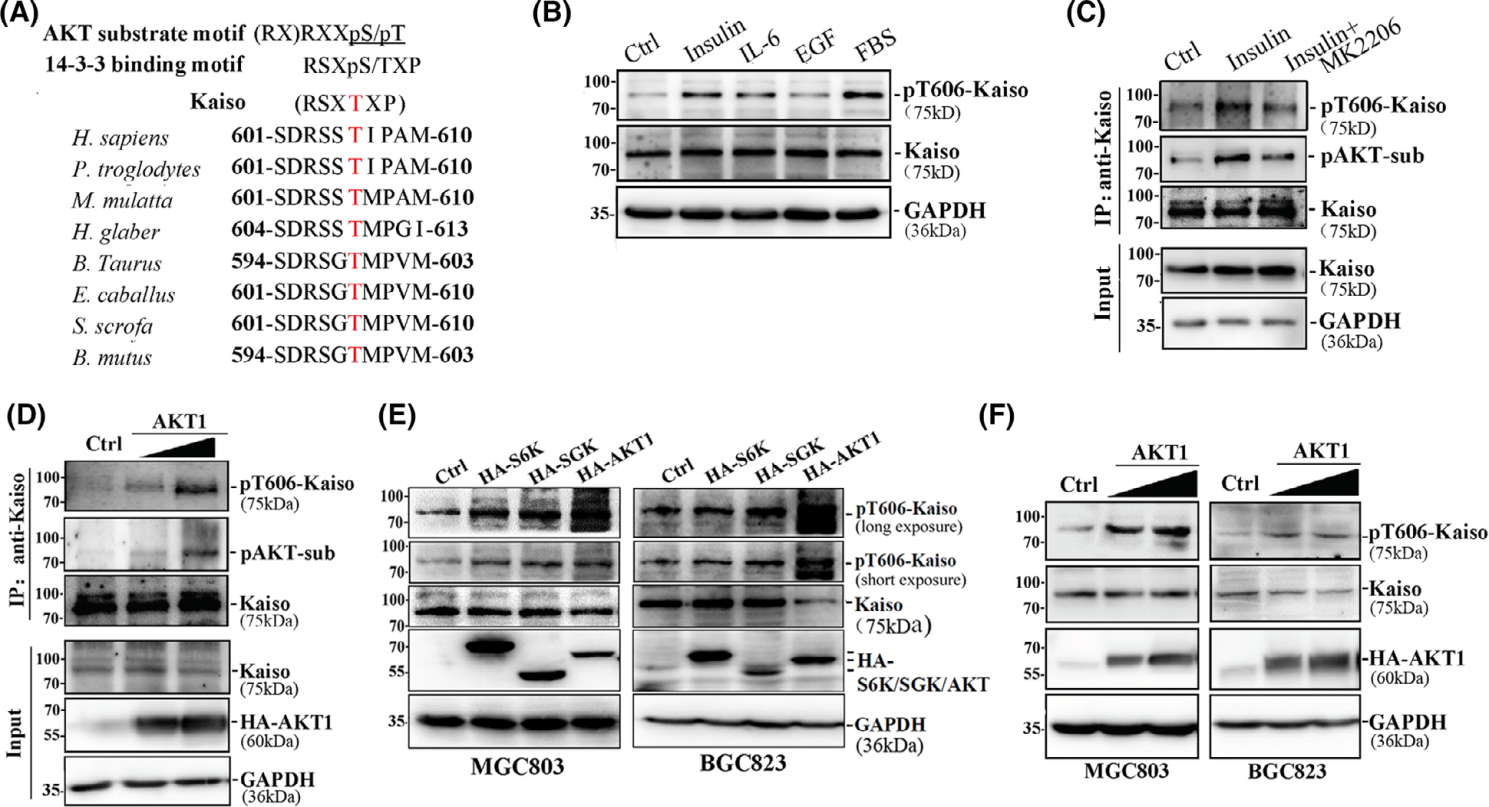
AKT1 increases the phosphorylation of Kaiso at T606. (A) A conservative RSXTXP motif within Kaiso of human and other species. (B) After starvation overnight, treatments of insulin (10 ng·mL^−1^), IL‐6 (1 ng·mL^−1^) and foetal bovine serum (FBS, 10% v/v) for 15 min increased the pT606‐Kaiso in MGC803 cells. The experiment was performed three times. (C) Effects of AKT inhibitor MK2206 treatment (10 μmol/mL) for 30 min blocked the promotion of insulin induced pT606‐Kaiso and Kaiso phosphorylation as AKT substrate (pAKT‐sub) in MGC803 cells. The experiment was performed two times. (D) *AKT1* overexpression at different doses increased the amount of pT606‐Kaiso and phosphorylated AKT substrate (pAKT‐sub) in Kaiso complexes immunoprecipitated by Kaiso antibody in MGC803 cells. The experiment was performed two times. (E) The activity comparison of three kinase candidates to phosphorylate Kaiso at T606 in MGC803 and BGC823 cells. The experiment was performed three times. (F) The T606‐phosphorylation status of endogenous Kaiso in MGC803 and BGC823 with *AKT1* overexpression after starvation overnight. The experiment was performed three times.

Human Kaiso contains a conservative RSSTIP motif with a Thr‐606 (T606) residue within the DNA‐binding motif (Fig. 2A; Fig. S1). Protein kinase B AKT1 is a typical kinase for the (RX)RXXpS/pT motif in multiple proteins such as mTOR, GSK‐3β, AMPKA and Catenin‐β1 [46, 47, 48, 49]. We speculated that the RSSTIP motif within Kaiso might be one of AKT1 targets.

To examine whether Kaiso was phosphorylated at T606 (pT606‐Kaiso) in human gastric cancer cells, a pT606‐Kaiso‐specific polyclonal antibody was generated from rabbit using the phosphorylated‐peptide LSD**RSSpTIP**AM as an antigen. The results of ELISA analysis employing the phosphorylated peptide LSD**RSSpTIP**AM and the non‐phosphorylated‐peptide LSD**RSSTIP**AM successfully verified that the anti pT606‐Kaiso antibody could specifically bind to phosphorylated Kaiso (Fig. S2A). The results of western blot analysis also showed that the pT606‐Kaiso‐specific antibody could bind to the overexpressed Kaiso‐WT, but not to the Kaiso‐T606A mutant (Fig. S2B).

To verify if Kaiso T606 could be a true phosphorylation site for the kinase AKT1, we stimulated the AKT activity in MGC803 cells with insulin, IL‐6, EGF and FBS after overnight starvation [50]. As expected, the phosphorylation level of endogenous Kaiso was markedly increased in MGC803 cells after insulin, IL‐6 and FBS stimulation, but not with EGF (Fig. 2B). Using the immunoprecipitation assay employing a phosphorylated‐(Ser/Thr) AKT substrate (pAKT‐sub)‐specific antibody, endogenous Kaiso signal could also be detected in the immunoprecipitated pAKT‐sub protein complexes, and vice versa (Fig. S3A). While the amount of total Kaiso was not affected by insulin, the amounts of both pT606‐Kaiso and pAKT‐sub in the Kaiso‐antibody‐immunoprecipitated protein complex were significantly increased by insulin stimulation and the increased pT606‐Kaiso and pAKT‐sub could be reversed by AKT inhibitor MK2206 treatment (Fig. 2C). A similar effect was also observed in MGC803 cells with *AKT1* overexpression (Fig. 2D).

We also tested the response of Kaiso to other AGC kinase members that prefer to target the same consensus motif (RxRxxS/T) [51] and found that, the activity of AKT1 to phosphorylate Kaiso at T606 was much higher than other two AGC kinases S6K and SGK in MGC803 and BGC823cells (Fig. 2E). As expected, the amount of pT606‐Kaiso was significantly increased in MGC803 and BGC823 cells with *AKT1* overexpression (Fig. 2F). Similarly, such effect was also detected in other cancer cell lines including MCF7, PC3, PANC1, but not in H1299 and RKO cell lines (Fig. S3B). Together, these results indicate that Kaiso can be phosphorylated at T606 by AKT1.

### 3.3 pT606‐Kaiso in the cytoplasm of human cancer cells and gastric mucosal tissues

**Fig. 3:**
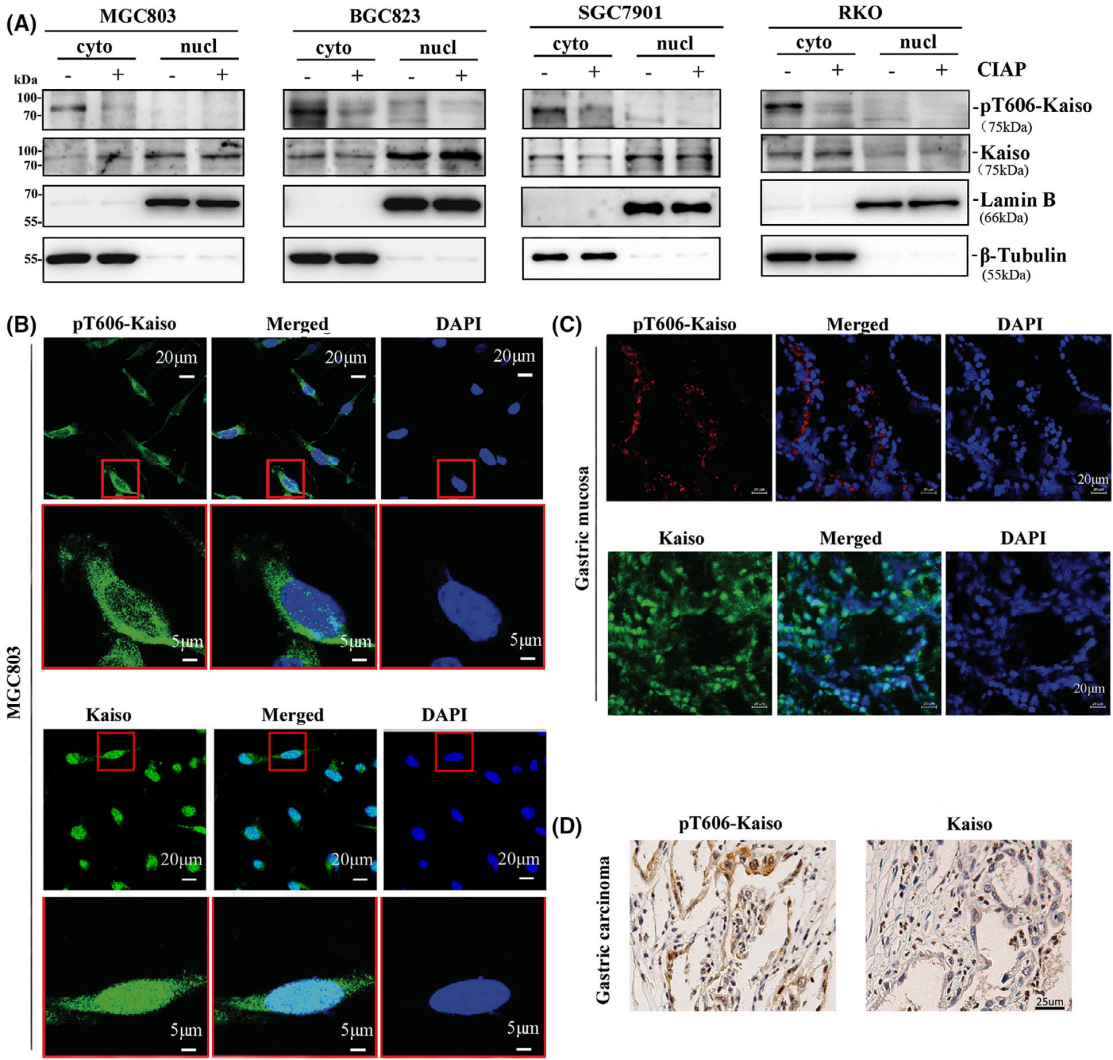
Subcellular localisation of pT606‐Kaiso in human cancer cell lines and gastric mucosal tissues. (A) Subcellular location of endogenous pT606‐Kaiso and total Kaiso in cytoplasmic and nuclear proteins (with and without de‐phosphorylation treatment by CIAP for 30 min) extracted from MGC803, BGC823, SGC7901 and RKO cell lines in western blotting. (B) Locations of pT606‐Kaiso and total Kaiso in the cytoplasm and nucleus in confocal microscopy analysis. (C) Locations of pT606‐Kaiso and total Kaiso in human gastric tissues in the confocal microscopy analysis. (D) Locations of pT606‐Kaiso and total Kaiso in representative gastric carcinoma tissues in IHC analysis. Scale bar: 20 or 5 μm in B, 20 μm in C, 25 μm in D. Gastric carcinoma and paired surgical margin samples from four patients were detected.

At the next step, to confirm the effect of the T606 phosphorylation on Kaiso compartmentalisation, the phosphorylation status of endogenous Kaiso in the nucleus and cytoplasm was evaluated using the pT606‐Kaiso specific antibody. In consistent with the results described above, most pT606‐Kaiso was detected in the cytoplasm of human cancer cells (MGC803, BGC823, SGC7901 and RKO) and the CIAP de‐phosphorylation treatment markedly decreased the amount of pT606‐Kaiso in the cytoplasm in western blotting (Fig. 3A). Using the anti‐pT606‐Kaiso‐specific antibody and the anti‐Kaiso commercial antibody, the confocal microscopy results further confirmed the cytoplasmic compartmentalisation of pT606‐Kaiso. Both cytoplasmic and nuclear compartmentalisation of Kaiso was observed in MGC803 cells (Fig. 3B). Similarly, in human gastric mucosal tissues, pT606‐Kaiso was only detected in the cytoplasm in the confocal microscopy and IHC analyses while total Kaiso was mainly detected in the nucleus (Fig. 3C,D). Collectively, the above results demonstrate that AKT1 can phosphorylate Kaiso at T606 and pT606‐Kaiso mainly locates in the cytoplasm of human cells.

### 3.4 pT606‐Kaiso interacts with 14‐3‐3 family members and accumulates in the cytoplasm

**Fig. 4:**
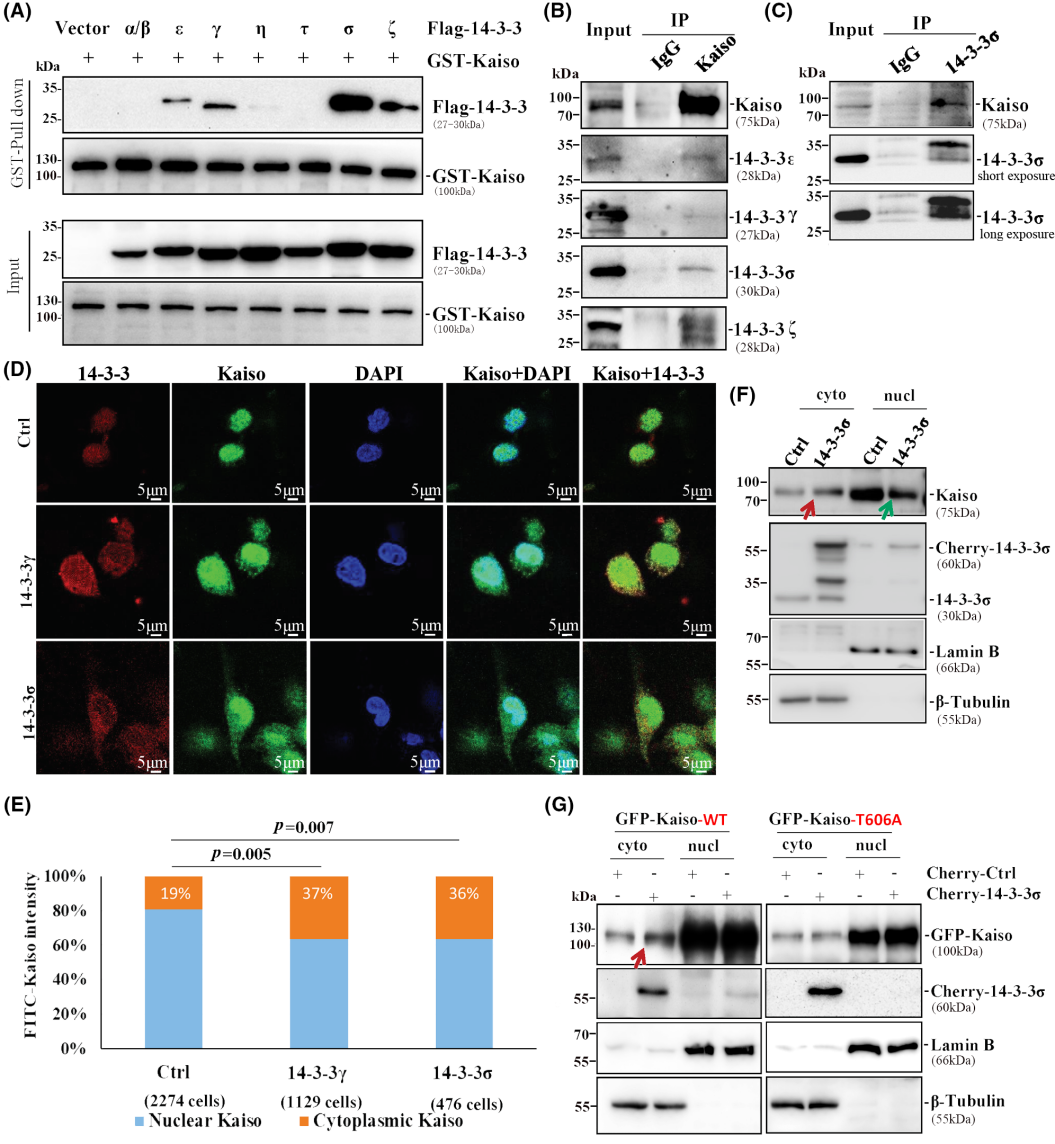
Kaiso interacted with 14‐3‐3 family members and accumulated in cytoplasm. (A) After the plasmids of 14‐3‐3 family members and GST‐Kaiso were co‐transfected into MGC803 cells, GST‐Kaiso pulled down various isoforms of 14‐3‐3 family, especially 14‐3‐3σ. The experiment was performed three times. (B) Endogenous Kaiso immunoprecipitated several 14‐3‐3 family members in MGC803 cell in Co‐IP analysis. (C) 14‐3‐3σ protein immunoprecipitated endogenous Kaiso in MGC803 cell in Co‐IP analysis. Co‐IP assay for the interaction of Kaiso and 14‐3‐3σ was performed three times. (D) The subcellular location of endogenous Kaiso in MGC803 cells with or without *14‐3‐3γ* or *14‐3‐3σ* overexpression by indirect immunofluorescence staining assay. As labelled, scale bar for IF = 5 μm. The experiment was performed three times. (E) Proportion of Kaiso in the nucleus and in cytoplasm of MGC803 cells with and without *14‐3‐3γ* or *14‐3‐3σ* overexpression. *N* = 2274/1129/476 in the group of Ctrl/14‐3‐3γ/14‐3‐3σ. *P*‐values in chi‐square tests are displayed. (F) Western blot for detecting the amounts of endogenous Kaiso in cytoplasm and nucleus protein in MGC803 cells with *14‐3‐3σ* overexpression. The experiment was performed three times. (G) Comparison of the levels of GFP‐Kaiso (wild‐type) or T606A mutant in the cytoplasm and nucleus in MGC803 cells with and without *14‐3‐3σ* overexpression. Red and Green arrows: makeable increased and decreased Kaiso in the cytoplasm and nucleus by 14‐3‐3σ. The experiment was performed three times.

It is well known that RSXpSXP is a 14‐3‐3 phosphoserine‐binding consensus motif [52]. To study whether the Kaiso RSSpTIP is a 14‐3‐3‐binding motif, we performed GST pull‐down and co‐immunoprecipitation (Co‐IP) experiments. The GST‐Kaiso pull‐down assay showed a strong pull down of 14‐3‐3σ (SFN), moderate pull‐downs of 14‐3‐3ε, 14‐3‐3γ, 14‐3‐3ζ and a weak pull‐down of 14‐3‐3η in MGC803 cell lysate (Fig. 4A). The Co‐IP results confirmed that endogenous Kaiso could bind to endogenous 14‐3‐3ε, 14‐3‐3γ, 14‐3‐3ζ and 14‐3‐3σ proteins in MGC803 cells (Fig. 4B), and that endogenous 14‐3‐3σ could also bind to endogenous Kaiso (Fig. 4C). In addition, *AKT1* overexpression, which promoted the phosphorylation of Kaiso at T606, further increased the Kaiso–14‐3‐3 interaction (Fig. S3C). These data indicate that Kaiso could interact with 14‐3‐3 family proteins in a T606 phosphorylation‐dependent manner.

It has been reported that intracellular Kaiso compartmentalisation is affected by growth conditions [14, 18]. Our confocal microscope analyses showed that 81% of endogenous Kaiso was observed in the nucleus of MGC803 cells (Fig. 4D,E). Notably, enforced mCherry‐14‐3‐3γ or ‐14‐3‐3σ overexpression significantly increased the proportion of endogenous Kaiso in the cytoplasm, compared to the mCherry control vector (from 19% to 37% or 36%) and Kaiso was mainly co‐localised with mCherry‐14‐3‐3γ or ‐14‐3‐3σ in the cytoplasm of these cells in the confocal analysis. Western blotting confirmed that mCherry‐14‐3‐3σ overexpression increased the cytoplasmic accumulation of endogenous Kaiso (Fig. 4F, red arrowed) while it decreased the nucleic Kaiso (Fig. 4F, green arrowed). In addition, 14‐3‐3σ only promoted the cytoplasmic accumulation of wild‐type Kaiso (Kaiso‐WT), but not the T606A mutant (Fig. 4G, red arrowed), implying an increase of pT606‐Kaiso in the cytoplasm by 14‐3‐3σ and likes. *14‐3‐3σ* or *14‐3‐3γ* overexpression also induced more cytoplasmic pT606‐Kaiso in MGC803 and BGC823 cell lines in western blotting (Fig. S3D,E). These results firmly confirm that the pT606‐Kaiso can interact with 14‐3‐3, which in turn accumulates the cytoplasmic Kaiso in human cancer cells.

### 3.5 The P120ctn interaction is essential for pT606‐Kaiso–14‐3‐3 accumulation in the cytoplasm

**Fig. 5:**
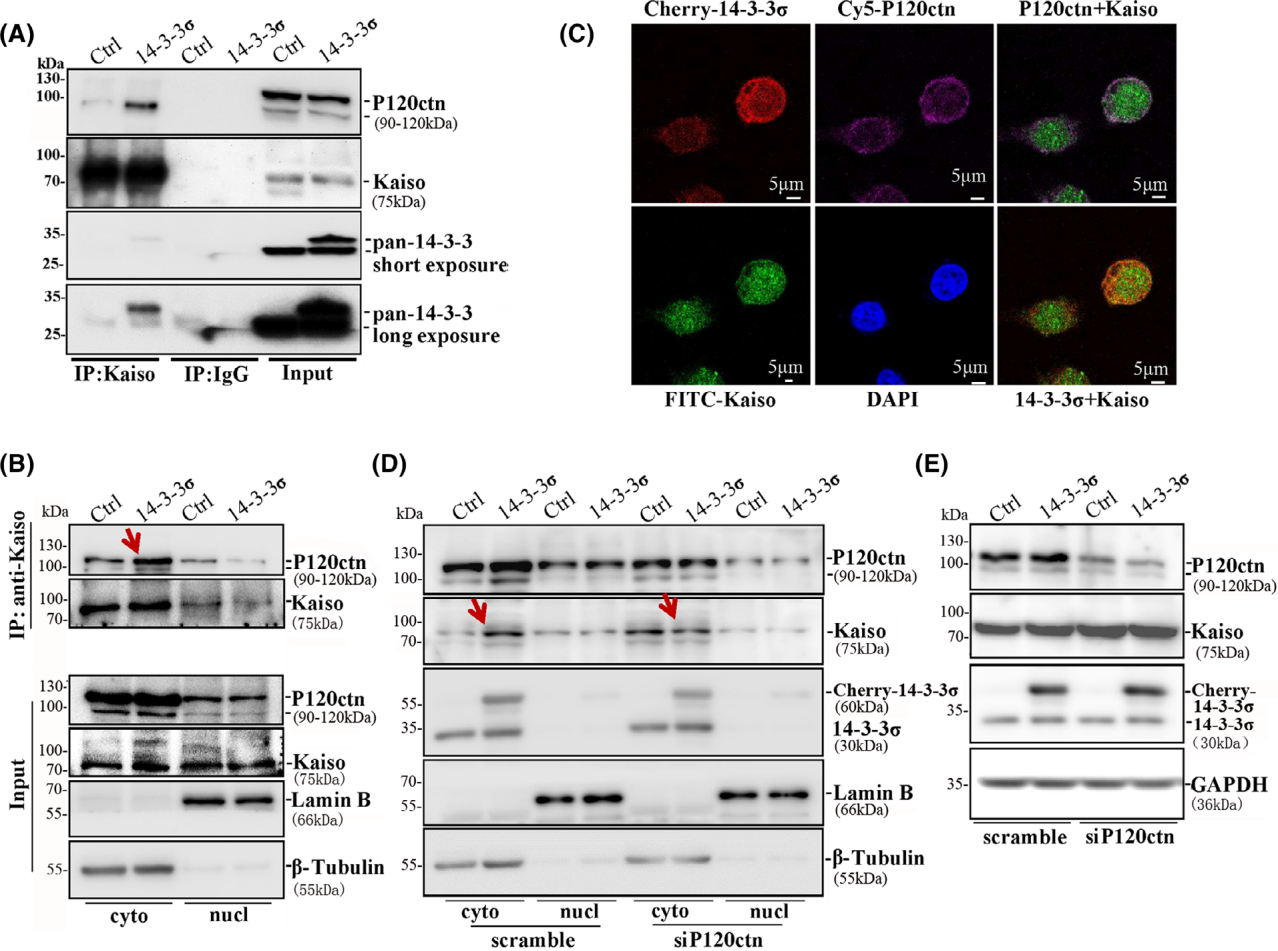
14‐3‐3σ promotes the interaction of Kaiso and P120ctn in the cytoplasm. (A) More Kaiso‐P120 complex was immunoprecipitated by Kaiso antibody in MGC803 cells with *14‐3‐3σ* overexpression in Co‐IP assay. Total protein levels of P120ctn and 14‐3‐3σ in the lysate of MGC803 cells with *14‐3‐3σ* overexpression were displayed in the right side. The experiment was performed three times. (B) Alterations of interactions between Kaiso and P120ctn proteins in the cytoplasm and nucleus of these cells with *14‐3‐3σ* overexpression in the Co‐IP assay using Kaiso antibody. The experiment was performed three times. (C) The subcellular locations of Kaiso, P120ctn and mCherry‐14‐3‐3σ in MGC803 cells. Scale bar: 5 μm. The experiment was performed two times. (D) Western blotting images for detecting effect of *P120ctn* knockdown and *14‐3‐3σ* overexpression on distribution of Kaiso in the cytoplasm and nucleus of MGC803 cells. The experiment was performed two times. (E) The levels of total P120ctn and Kaiso proteins in the lysate of MGC803 cells with *14‐3‐3σ* overexpression and siRNAs knockdown of *P120ctn* expression. The experiment was performed three times. Red arrow: P120ctn or Kaiso in the cytoplasm with and without makeable change.

As a given Kaiso interacting protein, the P120ctn binding is essential for Kaiso trafficking from the nucleus to the cytoplasm [19, 20]. Thus, we further studied whether 14‐3‐3 bound to the pT606‐Kaiso–P120ctn complex or bound to the pT606‐Kaiso alone in the cytoplasm. Interestingly, we found that *14‐3‐3σ* overexpression markedly increased the Kaiso‐interacted P120ctn, while the total levels of endogenous Kaiso and P120ctn proteins were not changed in MGC803 cells (Fig. 5A). Western blotting analysis also showed that endogenous P120ctn, AKT1 and 14‐3‐3σ proteins mainly located in the cytoplasm of MGC803 and BGC823 cells (Fig. S3F). Further analysis showed that more Kaiso–P120ctn binding was detected in the cytoplasm, but not in the nucleus of MGC803 cells with *14‐3‐3σ* overexpression (Fig. 5B). Immunofluorescence confocal microscopy showed that P120ctn, Kaiso and 14‐3‐3σ were mainly co‐localised in the cytoplasm (Fig. 5C). Moreover, when *P120ctn* was knocked down by siRNA (siP120ctn), the cytoplasmic Kaiso accumulation promoted by 14‐3‐3σ was diminished in the MGC 803 cells (Fig. 5D) while the level of total Kaiso was not changed (Fig. 5E). These results suggest that the P120ctn interaction is essential for the pT606‐Kaiso–14‐3‐3σ accumulation in the cytoplasm.

### 3.6 De‐repression of Kaiso target genes by T606 phosphorylation

**Fig. 6:**
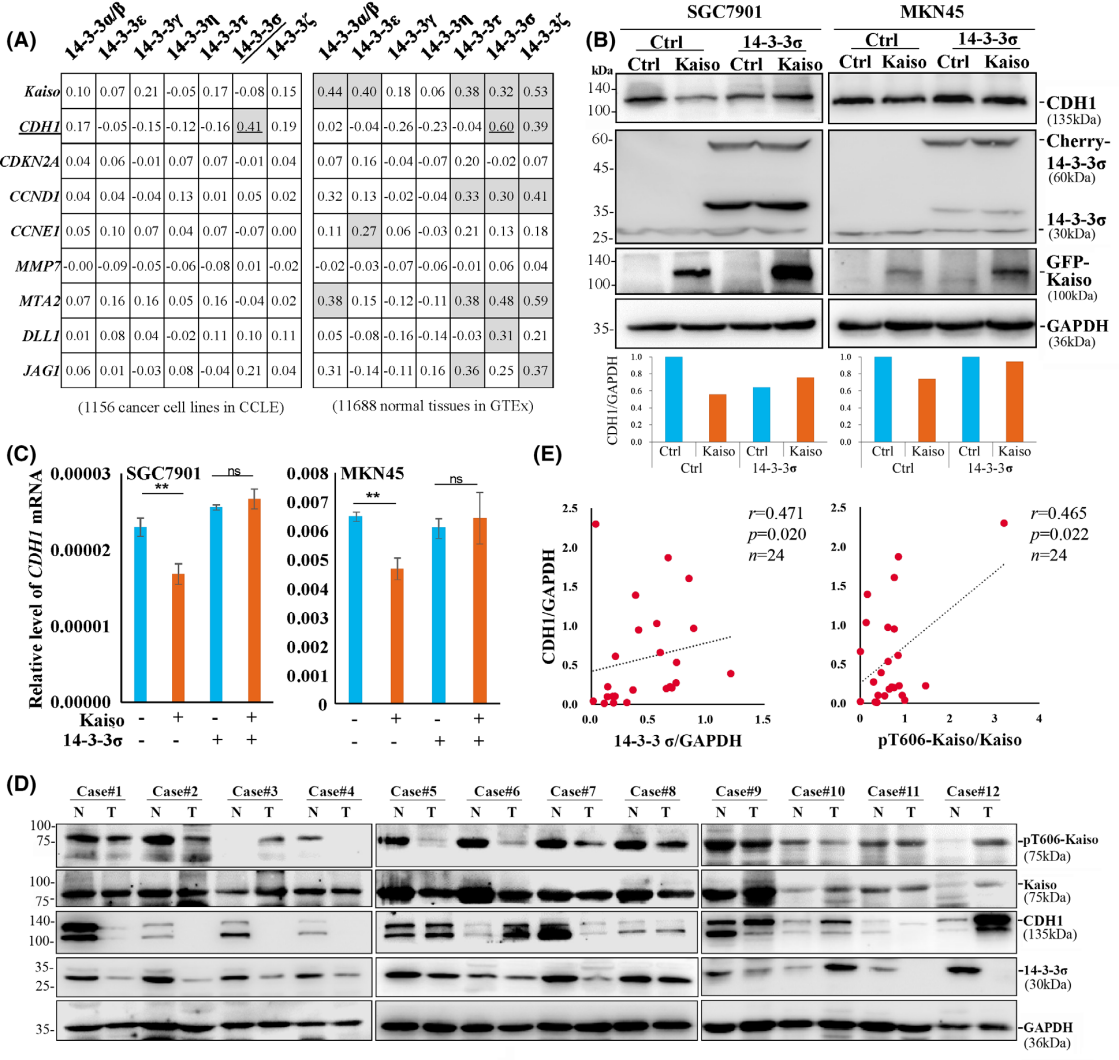
Effects of 14‐3‐3σ on inhibition of Kaiso's target gene *CDH1* expression. (A) The Pearson correlation coefficient between mRNA levels of 14‐3‐3 family members and Kaiso target genes in public RNA‐seq datasets from Cancer Cell Line Encyclopaedia (CCLE, 1156 cancer cell lines) and Genotype‐Tissue Expression (GTEx, 11 688 human normal tissues). (B) The level of *CDH1* in SGC7901 and MKN45 cells with or without wildtype Kaiso and *14‐3‐3σ* overexpression. The experiment was performed three times. (C) The level of the *CDH1* mRNA in SGC7901 and MKN45 cells with or without wildtype Kaiso and 1*4‐3‐3σ* overexpression (each experiment was repeated at least three times). Data were presented as the mean ± SD (*n* = 3). *P*‐values in Student's *t*‐test are labelled (**P < 0.01, ns: nonsense). (D) the amounts of pT606‐Kaiso, total Kaiso, 14‐3‐3σ and CDH1 proteins in gastric carcinoma (T) and the paired normal tissues (N) from 12 patients by western blotting. (E) Correlation between ratios of pT606‐Kaiso to total Kaiso and CDH1 to GAPDH proteins based on the density of these proteins in western blot. Spearman's rho coexpression coefficient (*r*) and *P*‐value are shown (*n* = 24).

It has been reported that Kaiso could bind to both methylated CGCG‐containing sequences and TpG‐containing specific binding sequences in target gene promoters and suppress their transcription [2, 3, 4, 5, 6]. We wondered whether T606 phosphorylation of Kaiso and its bindings to 14‐3‐3/P120ctn proteins could affect expression of Kaiso target genes through the cytoplasmic accumulation. By re‐analysing the RNA‐seq datasets for 14‐3‐3 family members and Kaiso target genes (including *CDH1*, *CCND1*, *CCNE1, MTA2, DLL1* and *DAG1*) from GTEx and CCLE [41, 42, 43, 44], we found that the mRNA level of 14‐3‐3σ mRNA was positively and most significantly correlated with that of *CDH1* in both normal human tissues (*n* = 11 688, *r* = 0.60, *P* < 0.001 in Pearson correlation analysis) in GTEx datasets and cancer cell lines in CCLE datasets (*n* = 1156, *r* = 0.41, *P* < 0.001) (Fig. 6A; Fig. S4A). This is consistent with the phenomenon that 14‐3‐3σ could strongly bind to Kaiso described above (Fig. 4A), suggesting an exact effect of 14‐3‐3σ expression on regulation of *CDH1* transcription, probably through the pT606‐Kaiso–14‐3‐3σ interaction. Interestingly, association between the mRNA levels of *CDH1* and *KAISO* was only observed in human normal tissue samples from subjects (*n* = 570) in the GTEx and most normal tissue samples from patients (*n* = 697) in the Cancer Genome Atlas (TCGA) datasets. The association between *CDH1* and *KAISO* was not found in cancer cell lines (*n* = 1063) in the CCLE datasets nor in cancer tissues of many organs in the TCGA datasets (Fig. S4B,C). These suggested that endogenous Kaiso may not be a repressor for *CDH1* transcription in normal cells via the translocation of phosphorylated Kaiso from the nucleus to the cytoplasm. But it may be a repressor for *CDH1* transcription in cancer cells because of blocks of the nucleus‐cytoplasm shift by the decreased level of Kaiso phosphorylation as described below.

Since we did not detect *CDH1* expression in MGC803 and BGC823 cells, two gastric cancer cell lines, SGC7901 and MKN45 with active *CDH1* expression were used to study the function of Kaiso as a transcription repressor and effects of 14‐3‐3σ expression changes on Kaiso target genes. As expected, Kaiso overexpression alone indeed decreased the amount of CDH1 whereas *14‐3‐3σ* co‐overexpression could abolish Kaiso‐induced CDH1 repression in SGC7901 and MKN45 cells in western blotting (Fig. 6B). These results were confirmed in qRT‐PCR analyses (Fig. 6C). A similar relationship was observed in gastric carcinoma tissues. While the level of pT606‐Kaiso was decreased in most gastric cancer samples, the ratio of pT606‐Kaiso to total Kaiso was positively and significantly associated with the amounts of CDH1 (adjusted by GAPDH) in gastric cancer and the paired normal tissue samples from 12 patients (Fig. 6D,E). Similar correlation was also found between CDH1 and 14‐3‐3σ. Taken together, the above results indicate that pT606‐Kaiso–14‐3‐3σ binding in the cytoplasm can deprive the function of Kaiso as a transcription repressor and lead to de‐repression of Kaiso target gene *CDH1*.

### 3.7 Tumour promotion by depriving pT606 phosphorylation of Kaiso

**Fig. 7:**
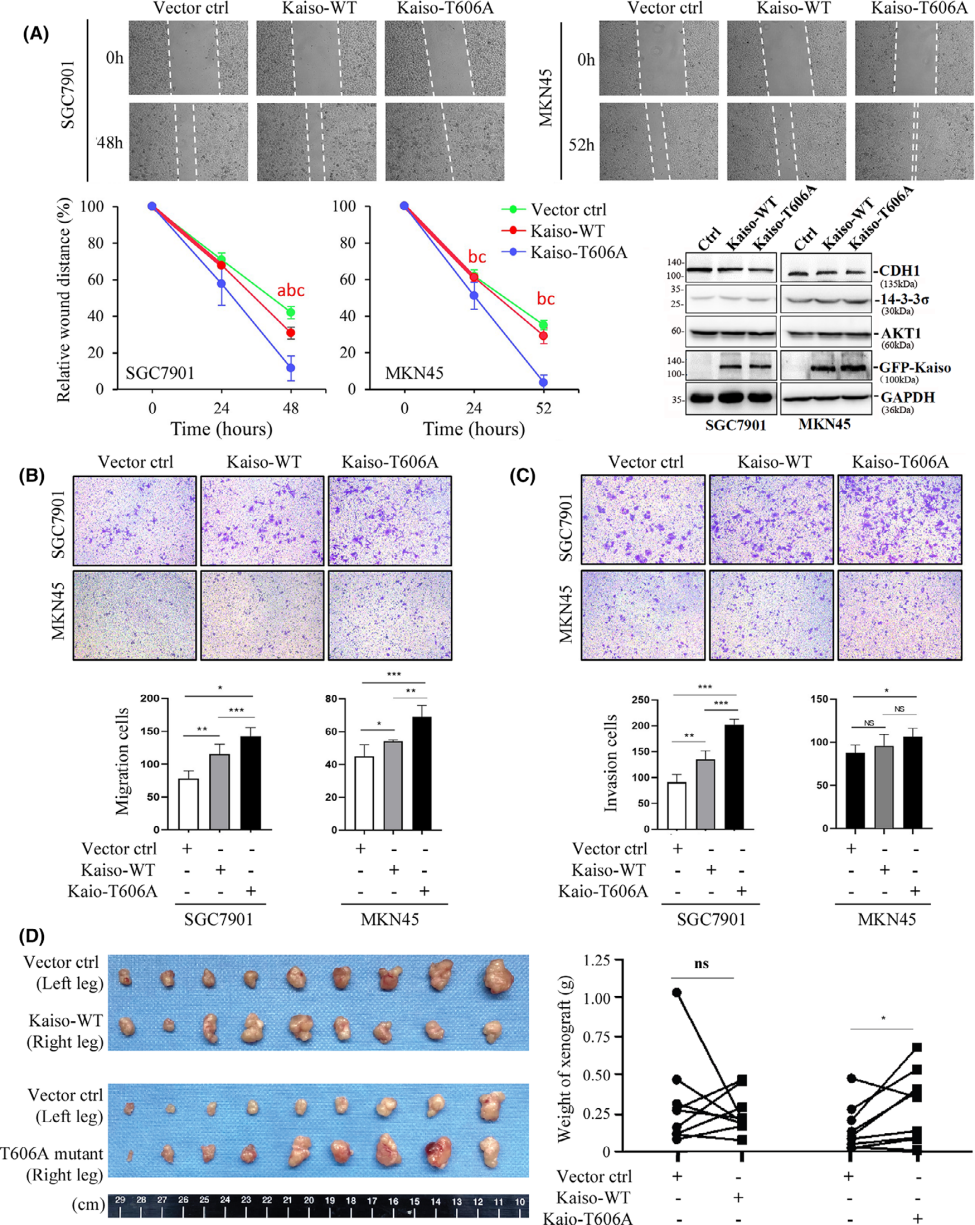
Effects of wild‐type Kaiso and its T606A mutant on gastric cancer cell migration and invasion *in vitro* and growth *in vivo*. (A) The percentage of open wounds was calculated to determine the migration of gastric cancer (GC) SGC7901 and MKN45 cells overexpressing Kaiso‐WT or its T606A mutant. The distance values are presented as mean ± SD (*n* = 4). a/b/c: *P* < 0.05 in Student's *t*‐test between Ctrl and Kaiso‐WT or Kaiso‐T606A and between Kaiso‐WT and Kaiso‐T606A; The results of western blot were also inserted to illustrate the status of Kaiso overexpression and CDH1 protein level; (B and C) The results of transwell assays to show the migration and invasion of GC cells overexpressing Kaiso‐WT or Kaiso‐T606A mutant respectively. Number of cells are presented as mean ± SD (*n* = 4); (D) Tumours derived from SGC7901 cells transfected with Kaiso‐WT and its T606A mutant or empty vector control. */**/***: *P* < 0.05/0.01/0.001 and *ns*: nonsense in Student's *t*‐test.

To evaluate the impact of pT606 phosphorylation of Kaiso on biological behaviours of cancer cells, we performed a set of functional assays through overexpression of wild‐type Kaiso or its T606A mutant that cannot be phosphorylated. A weak increase of gastric cancer cell proliferation was observed after overexpression of wild‐type Kaiso or T606A mutant comparing to the vector control (Fig. S5). In both wound healing and transwell assays, however, the cells migration was mostly increased by transiently overexpression of Kaiso T606A mutant while it was only weakly increased by transiently overexpression of wild‐type Kaiso in gastric SGC7901 and MKN45 cells (Fig. 7A,B). Similar differences were also observed in the invasion test (Fig. 7C). The animal experiments showed that the average weight of xenografts derived from SGC7901 cells stably transfected with Kaiso‐T606A mutant was significantly higher than that of cells transfected with empty vector control in NOD‐SCID mice (Fig. 7D, *P* < 0.05 in the paired *t*‐test). All these results showed that Kaiso T606A mutant significantly increased the migration and invasion of cancer cells *in vitro* and promoted the growth of cancer cells *in vivo* compared to wild‐type Kaiso.

## 4 Discussion

The subcellular locations of Kaiso determine its roles in normal cell differentiation and cancer development. However, detailed regulation machinery for the compartmentalisation of Kaiso is far from clear. In this study, we demonstrated for the first time that Kaiso could be phosphorylated at T606 by AKT1, the pT606‐Kaiso could interact with 14‐3‐3 and P120ctn in the cytoplasm and promote the shift of Kaiso from the nucleus to the cytoplasm. The phosphorylation of Kaiso finally leads to deprivation of transcription factor function of Kaiso and de‐repression of Kaiso target gene *CDH1* in normal tissues. Depletion of the T606 phosphorylation of Kaiso could augment repression of *CDH1* expression and promote cancer cell growth *in vivo*.

It is well known that 14‐3‐3 family proteins bind to common phosphoserine/phosphothreonine‐containing peptide motifs corresponding to Mode‐1 (RSXpSXP) or Mode‐2 (RXY/FXpSXP) sequences [47]. We found that Kaiso contains a very conservative RSSTIP motif that could be phosphorylated by AGC protein kinases, especially AKT1, in both *in vivo* and cell‐free system at RSSTIP‐T606 site. This is consistent with the report that T606 is one of phosphorylation sites of Kaiso in mass spectrometry analysis [22]. The pT606‐Kaiso could directly bind to 14‐3‐3 family proteins, and the T606A mutation could abolish most of Kaiso–14‐3‐3 binding, which indicates the T606 phosphorylation is essential for Kaiso–14‐3‐3 binding.

We previously reported that the neighbouring flank sequence (581‐631aa) of the zinc finger domain in the C‐terminus of Kaiso is required for its binding to *CDH1* promoter [53]. It was also reported that a region consisting of amino acid residues (454‐672aa) of Kaiso directly interacts with P120ctn [1] and Kaiso–P120ctn interaction promotes cytoplasmic‐nuclear trafficking of Kaiso [19, 20]. The phosphorylation site T606 is located within the P120ctn binding site, suggesting an effect of the phosphorylation at T606 on Kaiso–P120ctn interaction. In addition, P120ctn could bind to CDH1 and modulate its function and stability [54]. WNT‐stimulated P120ctn phosphorylation could promote P120ctn releasing from the CDH1–P120ctn complexes and enhancing the Kaiso–P120ctn interaction [55]. It is well known that most 14‐3‐3σ proteins localise in the cytoplasm [56] while Kaiso mainly localises in the nucleus [57]. Here, we observed that pT606‐Kaiso, 14‐3‐3 and P120ctn proteins could co‐localise in the cytoplasm and siRNA‐knockdown of *P120ctn* abolished the 14‐3‐3‐induced cytoplasmic Kaiso–14‐3‐3 binding. These results demonstrate that Kaiso–14‐3‐3σ interaction may be dependent on the Kaiso–P120ctn binding and the Kaiso, 14‐3‐3σ, and P120ctn may form a triplex in the cytoplasm. Since it was reported that cytoplasmic Kaiso functionally linked the autophagy‐related protein LC3A/B in breast cancer cells [58], how these proteins (Kaiso, 14‐3‐3σ and P120ctn) interact with each other is worth further studying.

Human *KAISO/ZBTB33* gene locates in chromosome X, which is frequently amplified in many types of cancers. It is controversy on the role of Kaiso in cancer development. For example, in the absence of the tumour suppressor *APC*, *Kaiso*‐deficient mice were resistant to intestinal cancer, suggesting that *Kaiso* might be an oncogene [16]. On the contrary, Kaiso has also been suggested to be a potential tumour suppressor, which repressed the transcription of *MMP7*, *CCND1* and *WNT11* genes involved in oncogenesis and metastasis [9, 11, 59]. Functions of Kaiso are tightly related to and dramatically influenced by microenvironmental factors [18]. We found here that the increased level of pT606‐Kaiso or pan‐14‐3‐3 (especially 14‐3‐3σ) expression could lead to Kaiso accumulation in the cytoplasm, deprivation of transcriptional factor function of Kaiso, and de‐repression of Kaiso target gene *CDH1*. Kaiso phosphorylation may account for the positive correlation between the levels of *ZBTB33/KAISO* and *CDH1* mRNAs in human normal tissues. In contrast, the decreased level or deprivation of pT606 phosphorylation of Kaiso could block the cytoplasmic transportation of Kaiso, repress *CDH1* transcription and promote the growth of cancer cells. Our results are consistent with the report that overexpression of *Kaiso/Zbtb33* resulted in downregulation of *Cdh1* in mice intestinal tissues [60]. Together, these phenomena indicate that Kaiso phosphorylation may be a crucial determinant for the roles of Kaiso in cancer development through de‐repression of tumour‐related genes.

## 5 Conclusion

Our studies reveal that Kaiso could be phosphorylated at T606 by AKT1 and pT606‐Kaiso accumulates in the cytoplasm through binding to 14‐3‐3/P120ctn, which de‐represses the Kaiso target gene *CDH1* in normal tissues. Decreased Kaiso phosphorylation might contribute to the development of gastrointestinal cancer.

## Conflict of interest

The authors declare no conflict of interest.

## Author contributions

DD, WT and SQ. designed research; WT, HY, SQ, and BZ performed research; DD, WL, LG and JZ analysed data; DD, WT and SQ wrote the paper.

### Peer review

The peer review history for this article is available at https://publons.com/publon/10.1002/1878‐0261.13292.

## Supporting information


**Fig. S1.** The status of Kaiso structure and protein modifications detected by LC/MS.
**Fig. S2.** Characterising the specificity of pT606‐Kaiso polyclonal antibody.
**Fig. S3.** AKT1 and 14‐3‐3 regulate the T606‐phosphorylation and subcellular localisation of endogenous Kaiso.
**Fig. S4.** Correlation between the levels of *CDH1* and *14‐3‐3σ* or *KAISO/ZBTB33* mRNAs in RNA‐seq and cDNA array datasets.
**Fig. S5.** Effect of wild‐type Kaiso and its T606A mutant on gastric cancer cell proliferation *in vitro*.

## Data Availability

The original contributions represented in the study are included in the article/Figs. S1–S5.
