# Supplementary material for: Kaiso phosphorylation at threonine 606 leads to its accumulation in the cytoplasm, reducing its transcriptional repression of the tumour suppressor CDH1
Source: Mol Oncol. 2022 Jul 28;16(17):3192–209. doi: 10.1002/1878-0261.13292 (PMC9441001; doi:10.1002/1878-0261.13292)
Supplement: Supplementary file 1 — Fig. S1. The status of Kaiso structure and protein modifications detected by LC/MS. Fig. S2. Characterising the specificity of pT606‐Kaiso polyclonal antibody. Fig. S3. AKT1 and 14‐3‐3 regulate the T606‐phosphorylation and subcellular localisation of endogenous Kaiso. Fig. S4. Correlation between the levels of CDH1 and 14‐3‐3σ or KAISO/ZBTB33 mRNAs in RNA‐seq and cDNA array datasets. Fig. S5. Effect of wild‐type Kaiso and its T606A mutant on gastric cancer cell proliferation in vitro. [file MOL2-16-3192-s001.docx]

**Supplemental Figures**

**
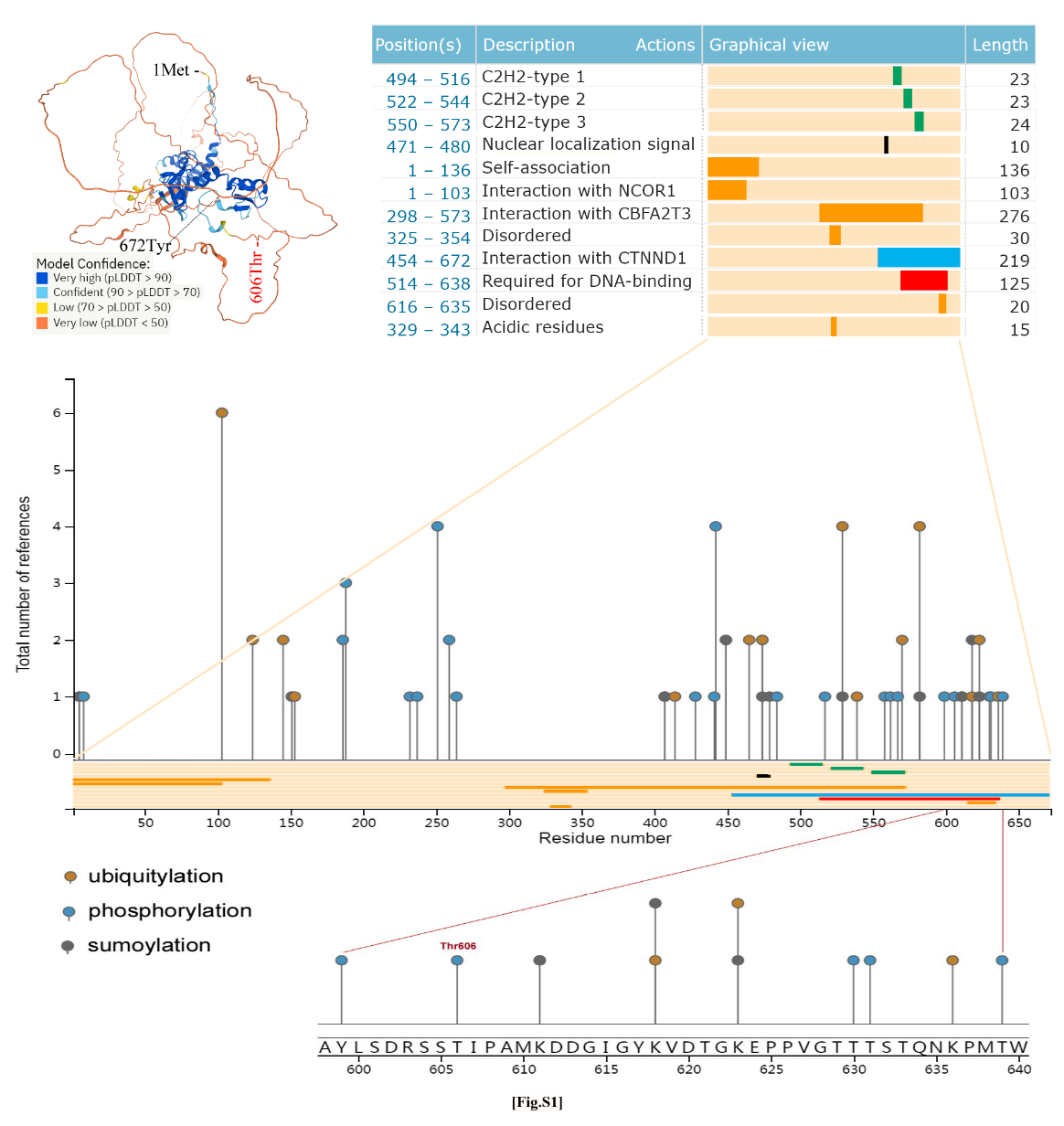
**

**Figure S1**. The status of Kaiso structure and protein modifications detected by LC/MS. Phosphorylation at the Thr606 residue in the LSDRSSTIPAM motif was illustrated in the bottom chart in details. The image was adapted with graphs for Kaiso modifications from the web site (www.phosphosite.org) (22). AlphaFold predicted 3D structures for Kaiso (ZBTB33/Q86T24) protein was adapted from images downloaded from the website (https://alphafold.ebi.ac.uk) (45); pLDDT, AlphaFold produced per-residue confidence score between 0 and 100. Information for Kaiso domains was adapted from images downloaded from the website (https://www.uniprot.org/uniprot/Q86T24) (1, 45).

**
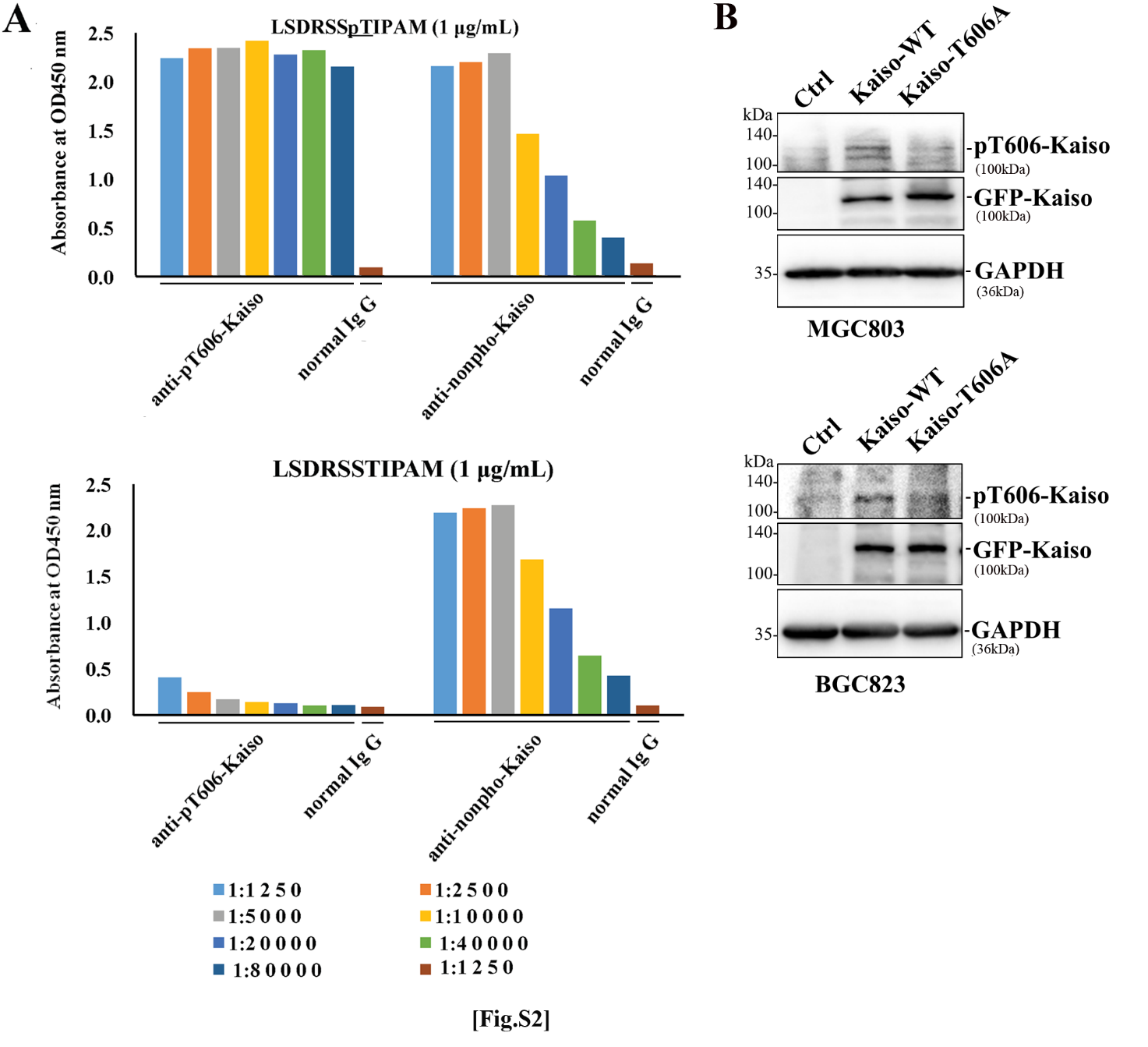
**

**Figure S2**. Characterizing the specificity of pT606-Kaiso polyclonal antibody. (**A**)ELISA results for the specificity of pT606-Kaiso and control antibodies against the pT606-Kaiso peptide (LSDRSSpTIPAM, the top chart) and for the specificity of pT606-Kaiso and control antibodies for the nonphosphorylated control peptide (LSDRSSTIPAM, the bottom chart). (**B**) Western blotting results for overexpressed GFP-Kaiso-WT and GFP-Kaiso-T606A with the prepared pT606-Kaiso polyclonal antibody.

**
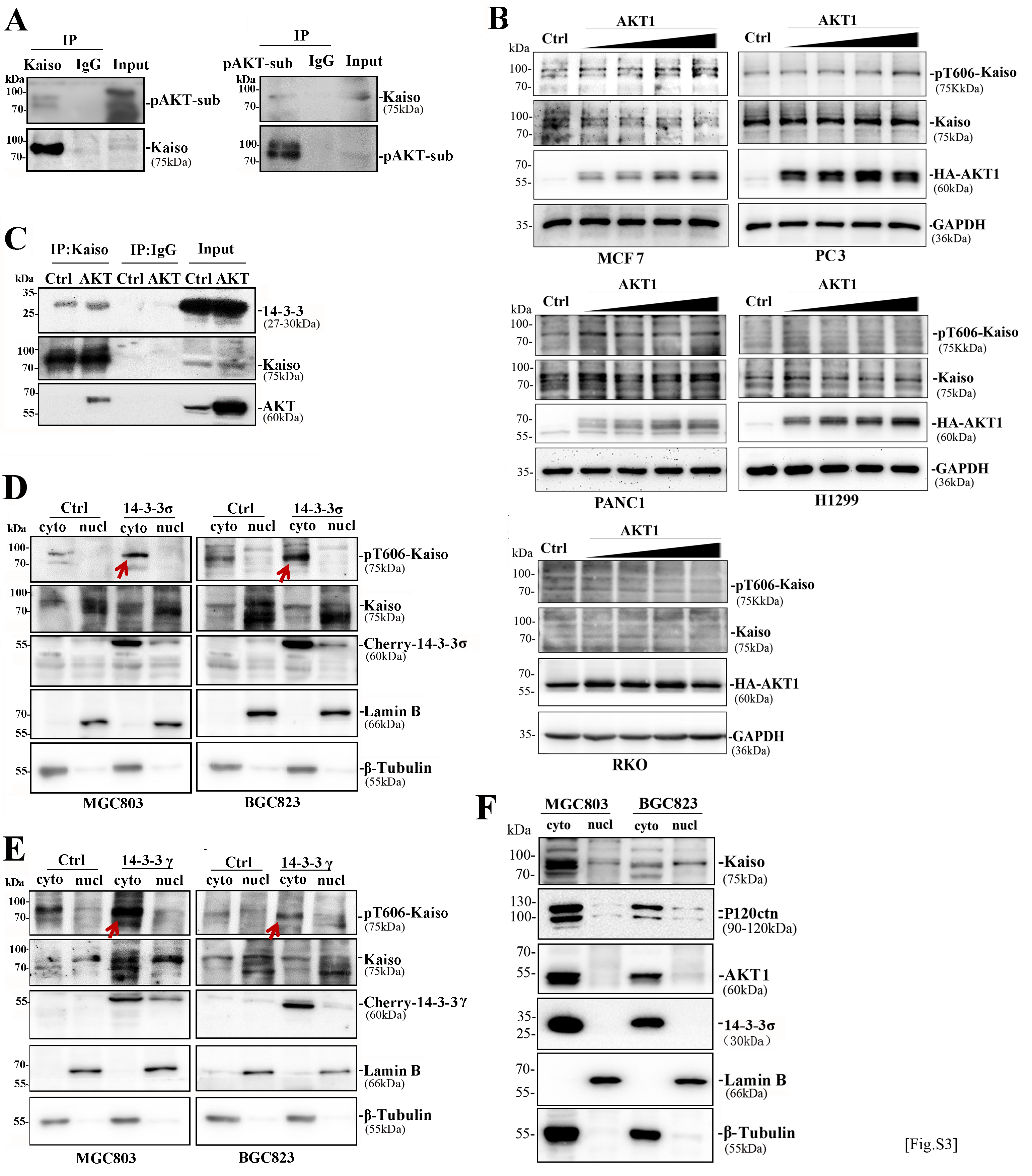
**

**Figure S3**. AKT1 and 14-3-3 regulate the T606-phosphorylation and subcellular localization of endogenous Kaiso. **(A**) Endogenous Kaiso in MGC803 cells immunoprecipitated by Kaiso antibody was identified by the antibody specific for AKT substrate motif, and the immunoprecipitation by AKT substrate antibody was identified by antibody against Kaiso. (**B**) The T606-phosphorylation status of endogenous Kaiso in MCF7, PC3, PANC1, H1299 and RKO cells with and without *AKT1* overexpression at different doses after starvation overnight. (**C**) *AKT1* overexpression increased endogenous Kaiso**‒**14-3-3 interaction in MGC803 cells in Co-IP assay. (**D** and **E**) The T606-phosphorylation states of endogenous Kaiso in the cytoplasm and nucleus of MGC803 and BGC823 cells with *14-3-3σ* and *14-3-3γ* overexpression, respectively. (**F**) Amounts of endogenous Kaiso, P120ctn, AKT1, and 14-3-3 σ in the cytoplasm and nucleus of MGC803 and BGC823 cells in Western blotting analysis.

**
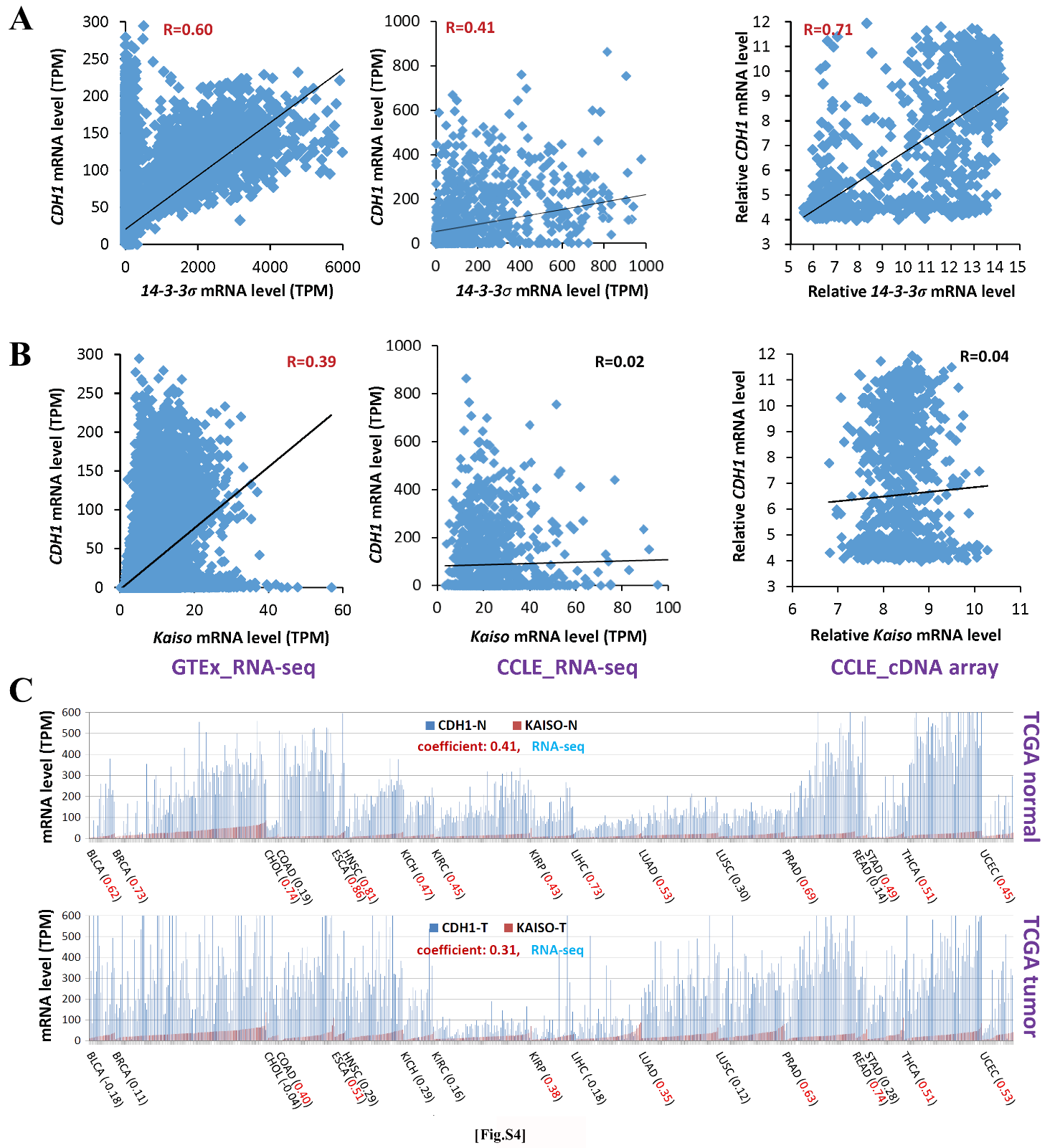
**

**Figure S4**. Correlation between the levels of *CDH1* and *14-3-3σ* or *KAISO/ZBTB33* mRNAs in RNA-seq and cDNA array datasets. (**A** and **B**) Human normal tissues in the Genotype-Tissue Expression (GTEx) project and cancer cell lines in Cancer Cell Line Encyclopedia (CCLE) project; (**C**) Human tumor tissue and the paired normal tissue samples from patients in the Cancer Genome Atlas (TCGA) project. Gene expression coefficient is labeled within parentheses for each kind of tissues.

**
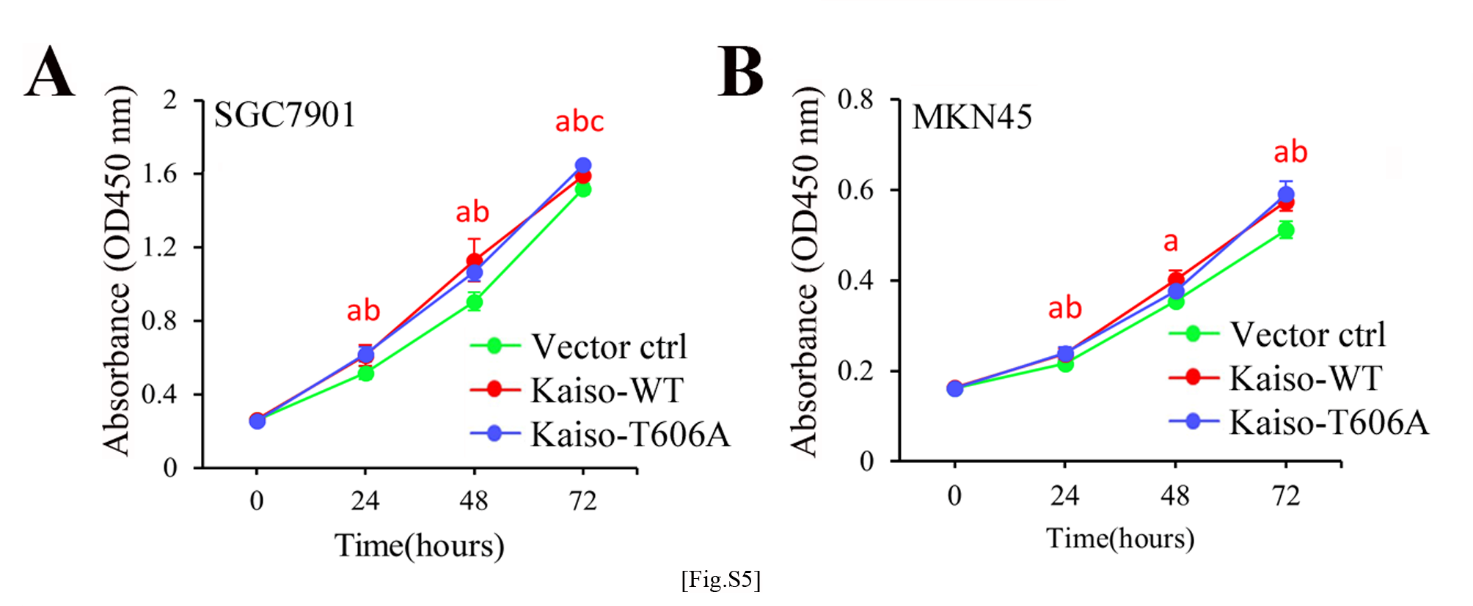
**

**Figure S5.** Effect of wildtype Kaiso and its T606A mutant on gastric cancer cell proliferation *in vitro.* (**A**) CCK-8 cell proliferation assay in SGC7901 cells stable overexpressed Kaiso-WT, Kaiso-T606A and vector control; (**B**) CCK-8 cell proliferation assay in MKN45 cells stable overexpressed Kaiso-WT, Kaiso-T606A and vector control. The absorbance values are presented as mean ± SD (n=4). a/b/c: *p* < 0.05 in Student's t-test between Ctrl and Kaiso-WT or Kaiso-T606A and between Kaiso-WT and Kaiso-T606A.
